# Serum Anti-Cryptosporidial gp15 Antibodies in Mothers and Children Less than 2 Years of Age in India

**DOI:** 10.4269/ajtmh.15-0044

**Published:** 2015-11-04

**Authors:** Robin P. Lazarus, Sitara S. R. Ajjampur, Rajiv Sarkar, Jayanthy C. Geetha, Ashok D. Prabakaran, Vasanth Velusamy, Elena N. Naumova, Honorine D. Ward, Gagandeep Kang

**Affiliations:** Division of Gastrointestinal Sciences, Christian Medical College, Tamil Nadu, India; Division of Geographic Medicine and Infectious Diseases, Tufts Medical Center, Tufts University School of Medicine, Boston, Massachusetts; Friedman School of Nutritional Science and Policy, Tufts University, Boston, Massachusetts

## Abstract

Little is known about the type and longevity of the humoral response to cryptosporidial infections in developing countries. We evaluated serum antibody response to *Cryptosporidium* gp15 in 150 sets of maternal, preweaning and postinfection/end-of-follow-up sera from children followed up to 2 years of age to determine the influence of maternal and preweaning serological status on childhood cryptosporidiosis. Fifty two percent (*N* = 78) of mothers and 20% (*N* = 30) of children were seropositive preweaning. However, most positive preweaning samples from children were collected early in life indicating transplacental transfer and subsequent rapid waning of antibodies. Although 62% (*N* = 94) of children had a parasitologically confirmed cryptosporidial infection (detected by stool polymerase chain reaction) during the follow-up, only 54% (*N* = 51) of children were seropositive postinfection. Given there were striking differences in seropositivity depending on when the sample was collected, even though *Cryptosporidium* was detected in the stool of the majority of the children, this study indicates that antibodies wane rapidly. During follow-up, the acquisition or severity of cryptosporidial infections was not influenced by maternal (*P* = 0.331 and 0.720, respectively) as well as the preweaning serological status of the child (*P* = 0.076 and 0.196, respectively).

## Introduction

*Cryptosporidium* is an important cause of gastroenteritis worldwide. In endemic regions, cryptosporidiosis is widely distributed within and across populations, ranging from self-limiting and/or asymptomatic infections in healthy people to life-threatening infections in immunocompromised individuals. Transmission of *Cryptosporidium* is predominantly through the fecal-oral route by the ingestion of oocysts, but can also occur by person-to-person contact and zoonotic infection.^1^,^2^ Individuals across all ages are affected, but in developing countries, the disease is seen predominantly in children where hygiene may be low and safe drinking water is scarce.^3^ The excretion of environmentally resistant oocysts into water sources results in contaminated water being a risk factor for cryptosporidiosis in industrialized countries.^4^–^6^ However, we have shown that provision of safe drinking water did not alter acquisition of infection or disease in young children in an urban slum in India,^6^ possibly indicating multiple modes of transmission in a contaminated setting.

Earlier studies on *Cryptosporidium* infections were based on screening by microscopic examination of stool samples.^7^ With the advent of molecular tools for detection of *Cryptosporidium* by polymerase chain reaction (PCR) at the small-subunit rRNA and at multiple other loci, the epidemiology, environmental sources, routes of transmission, genetic diversity, and parasite species–host dynamics have been more intensively studied.^8^–^11^

Serological assays based on the detection of *Cryptosporidium*-specific immunoglobulin G (IgG) identify more infections than conventional techniques such as microscopy or antigen detection.^12^–^14^ Cryptosporidial infection results in IgM-, IgG-, and IgA-specific serum antibody responses to the 17- (also called gp15)^15^ and 27-kDa (also called cp23)^16^ antigens of various *Cryptosporidium* subtypes and species.^17^–^20^ The antibody response after cryptosporidial infection appears to develop rapidly, peaking within 3–9 weeks and wanes to baseline levels by 5–6 months.^17^,^21^,^22^ Cell-mediated immunity is known to be important for protection from and resolution of cryptosporidial infections, but the role of antibody responses are not well understood.^23^,^24^ The humoral and interferon-γ-mediated cellular response induced by the gp15 (17 kDa) antigen of *Cryptosporidium* have been postulated to be protective,^25^ and therefore measuring antigen-specific cryptosporidial antibodies may be important in estimation of the protection conferred against disease by natural infection and reinfection in children. In addition, the role of maternal antibodies in susceptibility to infection during early childhood remains undefined.

This study was undertaken to determine the influence of the serological status of the mother on early childhood acquisition of cryptosporidiosis, the time to primary infection, and whether cryptosporidial antibodies in children could be used to predict risk of future infection or disease.

## Materials and Methods

### Study subjects and samples.

**Figure 1.**
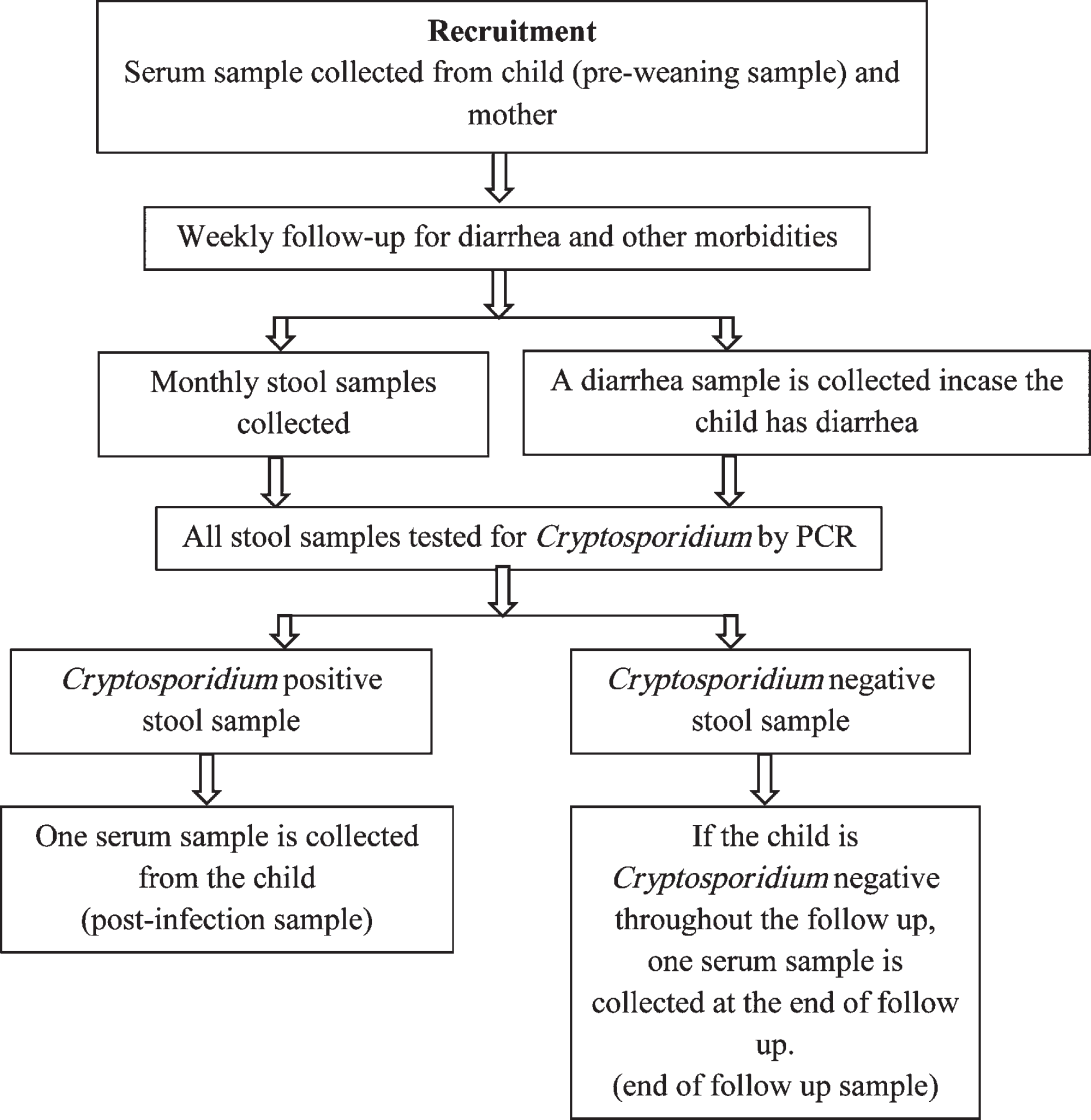
Follow-up and sample collection for 176 children recruited into a study on protection from cryptosporidial infection by bottled drinking water.

A total of 176 exclusively breast-fed children (defined as infants who received no food other than breast milk, either solid or liquid [including water], with the exception of oral rehydration solution or drops/syrups of vitamins, minerals, or medicines^26^) were recruited in a study investigating the protective efficacy of bottled water on childhood cryptosporidiosis in a semi-urban slum in Vellore, southern India.^6^,^27^ Based on the area of residence, families of the children received bottled (*N* = 90, protected) or municipal (*N* = 86, unprotected) drinking water, and the children were followed up until they attained 2 years of age; 160 (90.9%) of the 176 children completed the follow-up. Additional details of child recruitment and follow-up have been described previously.^27^ Surveillance stool samples were collected every month and diarrheal stool samples collected every time a child had an episode of diarrhea (defined as three or more loose, watery stools in a 24-hour period^28^). An infection was defined as symptomatic if a stool sample collected within ±7 days of a diarrheal episode was positive for *Cryptosporidium* spp. and asymptomatic if there was no diarrheal episode within a week before or after the detection of *Cryptosporidium* spp. in the stool sample.^6^ A blood sample was collected from mothers and exclusively breast-fed children at recruitment. In the event of a cryptosporidial infection, a blood sample was collected from the study subject as early as possible (not later than 6 months) after the first parasitologically confirmed infection (identified by stool PCR). At the end of 2 years of follow-up, a blood sample was collected from all children negative for cryptosporidiosis by fecal examination to ascertain missed cryptosporidial infections by serology (Figure 1

). The study was approved by the Institutional Review Boards of the Christian Medical College, Vellore, India, and Tufts University Health Sciences Campus, Boston, MA, and written informed consent was obtained from parents or legal guardians of all children before enrollment.

### Screening for *Cryptosporidium* spp.

All fecal samples were screened for *Cryptosporidium* spp. by 18S rRNA PCR on DNA extracted using a QIAamp Stool DNA Minikit (Qiagen Inc., Valencia, CA).^8^,^9^ In brief, this is a two-step nested-PCR followed by restriction fragment length polymorphism (RFLP) to identify *Cryptosporidium* species and genotypes. The PCR primers and cycling conditions and restriction enzymes have been described previously.^8^,^9^

### Enzyme-linked immunosorbent assay for anti gp15 IgG antibodies in serum.

Quantitation of serum IgG levels to the immunodominant gp15 antigen by enzyme-linked immunosorbent assay (ELISA) was carried out using recombinant (r)gp15 protein expressed in the pET46 vector (Novagen, EMD Biosciences, Inc., Merck KGaA, Darmstadt, Germany) as described previously^6^ and coated on a 96-well microtiter plate (Costar; Corning Inc., Corning, NY) overnight at 4°C in carbonate/bicarbonate buffer (pH 9.6) at a concentration of 0.5 μg of (r)gp15 protein/50 μL/well. Phosphate buffered saline (PBS) containing 0.05% Tween 20 was used to wash off excess antigen. To prevent nonspecific binding, the plates were blocked with 200 μL PBS containing 0.25% bovine serum albumin (BSA) (Sigma Aldrich, St. Louis, MO) for 2 hours at 37°C. Subsequently, 50 μL/well of serum samples diluted at 1:100 and 1:200 in PBS with 0.25% BSA along with a standard of pooled human IgG (Iviglob EX [5 g/100 mL]; VHB Life Science Ltd., Maharashtra, India) diluted serially from 1:50 to 1:3,200 was added to the wells. Wells at the periphery of the plate were used as blanks. Negative control sera (negative by ELISA and western blot analysis using *Cryptosporidium parvum* oocyst lysate as antigen) were run on each plate and incubated for 1 hour at 37°C. Plates were washed five times and 50 μL/well of alkaline phosphatase–conjugated goat antihuman IgG (γ-chain specific) (Sigma Aldrich) was added and incubated for 1 hour at 37°C. The plates were washed five times and 50 μL/well of substrate solution (100 mM Tris-HCl, pH 9.5, 100 mM NaCl, 5 mM MgCl_2_) containing 4-nitrophenyl phosphate disodium salt hexahydrate (1 mg/mL) (Sigma Aldrich) was added and incubated at room temperature for 15 minutes. The reaction was stopped with 50 μL/well of 0.1 M EDTA. Absorbance was measured at 405 nm using a microplate reader (ELX800; Biotek Instruments, Winooski, VT).^17^

The quantity of anti (r)gp15 IgG was determined by comparison of the optical density (OD) from sample wells to a standard curve generated by serial dilutions of pooled human IgG as described above. All samples and standards were run in duplicates, and the average OD of the blank wells in the periphery of the plate was subtracted. Each point on the standard curve was considered valid if the mean OD value was within a predetermined range and each plate was considered valid if at least 5 points of the standard curve were available and the mean negative control OD was < 0.1. The OD of all standards and samples was considered valid if the difference between the 2 replicates was < 0.1, and a coefficient of variation of < 15%. GraphPad Prism, Version 4.0, La Jolla, CA, was used to calculate unknown values for the samples from the linear part of the sigmoidal dose–response curve. The value obtained was multiplied by the dilution factor of 1 and 2 for 1:100 and 1:200 dilutions, respectively, and the results were expressed as arbitrary units (AU). The mean AU of 1:100 and 1:200 dilutions for each sample was calculated and considered valid if they had a coefficient of variation of < 15%.

Any sample with a detectable antibody level was considered seropositive. The proportions of seropositive maternal, pre- and postinfection samples were calculated along with geometric mean concentrations (GMCs) and 95% confidence intervals (95% CIs). Children were considered to have seroconverted if they were seronegative preweaning, but became seropositive postinfection or at the end of follow-up.

### Statistical analysis.

Data were entered in duplicate using Epi-Info 2002 (CDC, Atlanta, GA). The two entry datasets were compared to detect missing values or discrepancies between them, and identical values were saved to a master database to be used for all subsequent analyses. The data analysis was performed in STATA 10.1 for Windows software (StataCorp, College Station, TX). Continuous variables were compared using two-tailed *t* test if normally distributed or using Mann–Whitney *U* test if the distribution was skewed. Categorical variables were compared using χ^2^ test if the expected cell count was ≥ 5 or using Fisher's exact test if the expected cell count was < 5.

## Results

Of the 160 children who completed the follow-up until 2 years of age, a complete set of maternal, preweaning and postinfection (*N* = 94), or end- of-follow-up (*N* = 56) serum samples as per protocol were available for 150 (94%). Children for whom the complete set of serum samples were available were more likely to belong to a joint or an extended family (*P* = 0.044), had a larger family size (*P* = 0.017), and were exclusively breast-fed for a longer duration of 4.8 (3.6–5.7) months compared with children with an incomplete set of serum samples (3.5 (1.9–5.3) months, *P* = 0.029). Other sociodemographic characteristics such as gender, socioeconomic status, presence of toilet, and animals at home and household hygiene were comparable between children with and without a complete set of serum samples (Table 1). There was no difference in the proportion of children with complete sets of serum samples between the bottled (73/90, 81%) and municipal (77/86, 90%) water groups (*P* = 0.115).

### Comparison of maternal and preweaning antibody levels.

Of the 150 maternal samples, 78 (52%) were seropositive with a GMC of 465.6 (95% CI = 391.3–554) AU and 72 (48%) were seronegative. The proportion of seropositive mothers was comparable between the bottled (58%) and municipal (47%) water groups (*P* = 0.187), as were the GMCs (524.1 versus 405.2 AU, *P* = 0.092) (Table 2).

Among the 150 children, 30 (20%) preweaning samples were seropositive with a GMC of 342.7 (95% CI = 289.0–406.4) AU and 120 (80%) were seronegative. There was no significant difference between proportion of children seropositive preweaning in the bottled (26%) and municipal (14%) water groups (*P* = 0.072). The preweaning GMCs were also comparable between children in the bottled and municipal water groups (315.9 versus 394.6 AU, *P* = 0.254) (Table 2).

The median (interquartile range [IQR]) age of children at the time of collection of the preweaning sera was 34 (13–65) days. It was 34 (10–67) and 33 (13–63) days among children in the bottled and municipal water groups, respectively (*P* = 0.891). However, children who were seropositive had their samples collected at an earlier age (median [IQR] = 4 [1–20] days) than those who were seronegative (42 [18–70] days, *P* < 0.001). This trend was similar among children in the bottled (4 [1–37] versus 46 [18–79] days, *P* = 0.002) as well as the municipal (3 [1–13] versus 40 [18–66] days, *P* < 0.001) water group.

When the mother–child paired sera were considered, 21 (26.9%) children of the 78 seropositive mothers were seropositive with a GMC of 350.3 (95% CI = 281.0–436.6) AU, whereas only 9 (12.5%) children of the 72 seronegative mothers were seropositive with a GMC of 326 (95% CI = 238.4–445.6) AU, indicating a significant association between the presence of antibodies in mothers and their children preweaning (*P* = 0.027). A dose–response relationship between maternal antibody levels and the likelihood of a child being seropositive preweaning, was also noticed (Table 3).

### Serological response to cryptosporidial infections.

Sixty two (41%) of the 150 children with complete sample sets were seropositive in their postinfection/end-of-follow-up samples. There was no difference between proportion of seropositive children, postinfection, in the bottled (42%) and the municipal (40%) water groups (*P* = 0.784). The postinfection/end-of-follow-up GMCs were also comparable between children in the bottled and the municipal water groups (609.3 versus 646.8 AU, *P* = 0.667) (Table 2).

During their 2-year follow-up period, 94 (62%) of 150 children had a parasitologically confirmed cryptosporidial infection; of them 51 (54%) also had detectable antibodies in their postinfection serum samples with a GMC of 666.2 (95% CI = 538.1–824.6). In addition, 11 (20%) of the 56 children who did not have a parasitologically confirmed cryptosporidial infection had detectable antibody levels in their end-of-follow-up serum sample with a GMC of 476.8 ((95% CI = 290.8–781.7). The postinfection/end-of-follow-up GMCs were comparable between children with and without a parasitologically confirmed cryptosporidial infection (*P* = 0.159).

The median (IQR) duration between a parasitologically confirmed cryptosporidial infection and the collection of postinfection serum sample was 38 (23–64) days. Children who were seropositive had their postinfection serum samples collected earlier (29 [21–50] days post detection) than seronegative children (50 [24–96] days post detection, *P* = 0.041).

### Maternal and preweaning serological status and cryptosporidial infections.

There was no difference between seropositive and seronegative mothers in the proportion of children who were stool positive for *Cryptosporidium* spp. (46/78, 59% children of seropositive mothers and 48/72, 67% children of seronegative mothers, *P* = 0.331). There was no difference in the postinfection/end-of-follow-up seropositivity between children of seropositive and seronegative mothers (47% versus 35%, *P* = 0.114).

Among the 120 children who were seronegative preweaning, 71 (59%) developed a parasitologically confirmed cryptosporidial infection during the follow-up, compared with 23/30 (77%) seropositive children (*P* = 0.076). The postinfection/end-of-follow-up seropositivity status was comparable in children with or without a positive preweaning sample (47% versus 40%, *P* = 0.507). Interestingly, however, all 11 children with parasitologically negative but serologically positive cryptosporidial infection were seronegative preweaning, indicating seroconversion following an infection missed by the study sampling.

### Severity of infection and serological status in mothers and children.

Of the 94 children with cryptosporidiosis, 67 (71%) had an asymptomatic first infection, whereas 27 (29%) had diarrhea with the first infection. There was no difference in seropositivity between children who had asymptomatic (39/67, 58%) or symptomatic (12/27, 44%) infections (*P* = 0.258). Seropositive children with asymptomatic infection had higher gp15 IgG levels (752.6, 582.7–972.0) postinfection than those with symptomatic infections (448.1, 327.9–612.2), but this difference was not statistically significant (*P* = 0.060).

When the severity of infections were analyzed by maternal serological status, children of seronegative and seropositive mothers were equally likely to have a symptomatic infection (48% versus 52%, *P* = 0.720). However, children with a negative preweaning serum sample were almost twice as likely to have cryptosporidial diarrhea as children with a positive sample, although this difference was not statistically significant (32% versus 17%, *P* = 0.196).

## Discussion

Antibodies to gp15 were evaluated within a quasi-experimental study investigating the protective efficacy of bottled and municipal water against cryptosporidial infections in children < 2 years of age.^6^,^27^ There are very few studies that have examined sera for antibodies against cryptosporidial proteins at multiple time points in children in whom longitudinal follow-up, with or without stool sampling for *Cryptosporidium*, has been undertaken. This study recruited exclusively breast-fed children and demonstrated a high correlation between seropositivity of mothers and their children in samples collected before weaning, indicating efficient transplacental transfer of antibodies as had been described in Brazilian infants,^29^ but differing from data in Bedouin infants where it was estimated that about one-third of the cryptosporidial antibodies were transplacental.^30^ However, the antigens used in the three studies were different, with cp23 antigen in Brazil, calf oocyst lysate in Bedouin infants, and gp15 in this study (Table 4). The gp15 and cp23 are sporozoite-derived antigens that are considered specific and immunodominant.^14^,^17^,^29^ It would be interesting to estimate and compare the two immunodominant antigens (gp15 and cp23) to better understand the serological response to cryptosporidial antigens, transplacentally and after an infection.

**Figure 2.**
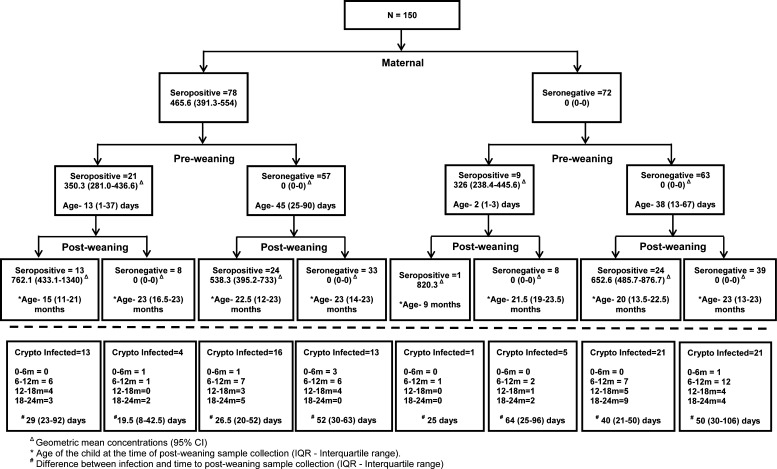
Flowchart showing the serological status of mothers and children (pre- and postweaning). Infections identified by stool polymerase chain reaction (PCR) or serology are shown below the dashed line for each category.

Previous studies have shown that most maternally acquired antibodies fall to low levels by the 6th month of life, rendering 60–80% of children seronegative.^29^,^30^ Also, antibody responses to cryptosporidial gp15 antigen have been predicted to peak at approximately 3–9 weeks after an episode of cryptosporidial diarrhea and wanes to baseline levels by 5–6 months.^17^,^21^,^22^ In this study, however, only one-fourth of children of seropositive mothers had detectable antibodies in their preweaning samples, although they were collected within the first 4 months of birth. Moreover, only 54% children with a parasitologically confirmed cryptosporidial infection had detectable antibodies postinfection, despite samples being collected within 9 weeks of infection (Figure 2

). Striking difference in seropositivity was noticed depending on when the preweaning or the postinfection sample was collected. Children in whom preweaning/postinfection samples were collected earlier were more likely to be seropositive. Taken together, these observations suggest that cryptosporidial antibodies possibly wane much faster than what was previously believed. A half-life of 12 weeks has been estimated for antibodies to the 27- and 17-kDa (gp15) cryptosporidial antigens in adults^12^; but there is no information on the half-life of the anti-gp15 IgG antibodies in children in developing countries and whether it differs from that in adults.

After the loss of maternal or preweaning antibodies, about half of the study children who were exposed to cryptosporidial antigen did not develop or sustain an immune response detectable in the postinfection or end-of-follow-up sample. The lack of detection of antibodies might have been either because no immune response was mounted by the host, with *Cryptosporidium* detection by PCR not indicating replicating parasites, but presence in the gut possibly as part of an extremely contaminated environment, or because the immune response was short lived and the timing of sample collection was too late to detect antibodies. The presence of cryptosporidial antibodies might be expected to prevent binding of *Cryptosporidium* to the intestinal epithelium and therefore replication in the gut.^15^,^23^,^33^,^39^

At the end of 2 years, 41% of children were seropositive in this study, which was similar to the study in a Brazilian peri-urban area where 41.2% seropositivity was seen in children aged between 0 and 4 years.^40^ Several other studies have reported seropositivity to *Cryptosporidium* ranging from 46% to 89%,^29^,^30^,^36^,^40^–^44^ and this may depend on several factors involving the level of exposure affected by sanitation and hygiene, drinking water, food, socioeconomic status, environmental conditions and animal exposure, inter-study and population differences and, more importantly, variations in the ELISA procedures (cryptosporidial antigens [crude oocysts versus sporozoite antigens], serum dilutions, and methods used in the calculation of antibody titers) (Table 4). Previous studies in the same study area used (r)gp15 that was expressed in the pET 32/Xa/LIC vector (Novagen, EMD Biosciences, Inc., Merck KGaA, Darmstadt, Germany), which encodes a fusion tag including thioredoxin, S-tag, and His tag.^17^,^37^ To control for the possibility of nonspecific antibody responses to the fusion tags, a recombinant control protein containing only the fusion was also used, and the OD from the control protein was subtracted from that of the (r)gp15 protein and a semiquantitative assay with antibody levels calculated based on OD values was used. By contrast, this study used (r)gp15 expressed in the pET46 vector (Novagen, EMD Biosciences, Inc., Merck KGaA, Darmstadt, Germany), which encodes only a 6-His tag. Since human sera did not react with the His tag alone, a control protein was not used. Compared with the previous study, using this modified assay there were marked differences with 94.6% of children seropositive by 2 years of age in the earlier study compared with 41% in this study. Maternal seropositivity was also low at 52% in this study, compared with 89% in the previous study.^37^ It is probable that nonspecific antibody responses were reduced significantly by using the 6-His tag–encoded(r)gp15 antigen, therefore resulting in a more specific quantitative assay for measuring gp15 antibody levels.

In this study, the proportion of cryptosporidial infections was slightly higher in children of seronegative mothers compared with children of seropositive mothers (67% versus 59%). Also, children who were seronegative preweaning were slightly more susceptible to symptomatic infections than seropositive children (32% versus 17%). Although not a marked effect, these observations might indicate partial protection by preexisting antibodies.^39^ Higher antibody levels in asymptomatic compared with symptomatic children could be due to the booster effect of secondary exposures to the cryptosporidial antigen.^33^,^38^

In conclusion, our study demonstrated serological evidence of frequent exposure to *Cryptosporidium* spp. in early life, which may persist into adulthood, as demonstrated by the acquisition and increase in antibodies in a majority of children and the finding of antibodies to gp15 in mothers. However, the maternal antibody status did not influence acquisition or severity of infection or antibody levels in children. Antibodies also appeared to wane rapidly in children, but because there was limited follow-up of children after the second blood sample, the level of protection from infection or disease in children with and without antibodies cannot be determined. The modified assay used in this study demonstrated a more specific antibody response toward the cryptosporidial gp15 antigen. Prospective future studies with longitudinal sampling methods that combine parasitological and serological data would enable us to better understand the correlates of protection against cryptosporidial infections.

## Figures and Tables

**Table 1:** Comparison of baseline characteristics between children who had complete sets of serum samples (*N* = 150) and those who did not (*N* = 26)

	Children with complete set of serum samples	Children without complete set of serum samples	*P* value
Male child	83 (55%)	12 (46%)	0.386‡
Median (IQR) birth weight (in kg)*	2.9 (2.7–3.1)	2.9 (2.5–3.3)	0.806§
Median (IQR) family size	6 (4–7)	5 (4–6)	0.017§
Nuclear family	66 (44%)	17 (65%)	0.044‡
Crowded living conditions (≥ 5 per room)	58 (39%)	6 (23%)	0.127‡
Presence of older sibling(s)	104 (69%)	15 (58%)	0.242‡
Median (IQR) age of the mother (in years)	24 (21–26)	25 (23–26)	0.146§
Median (IQR) years of completed maternal education	7 (3–10)	8 (6–10)	0.162§
Living in a “kutcha” house†	28 (19%)	6 (23%)	0.599‡
Median (IQR) duration of exclusive breast-feeding (in months)	4.8 (3.6–5.7)	3.5 (1.9–5.3)	0.029§
Low socioeconomic status	99 (66%)	18 (69%)	0.747‡
Firewood as the primary cooking mode	78 (52%)	12 (46%)	0.582‡
Presence of toilet in the house	92 (62%)	10 (63%)	0.953‡
Presence of animal(s) in the house	44 (29%)	6 (23%)	0.514‡
Good household hygiene	41 (27%)	6 (23%)	0.651‡

IQR = interquartile range

* Data on birth weight and presence of toilet missing for 9 and 11 children, respectively.

† “Kutcha” house: a house with wall and roof of mud/tin/asbestos/thatch.

Tests of significance: ‡χ^2^ test; §Mann–Whitney *U* test.

**Table 2:** Proportion of seropositive mothers and children at the start and end of follow-up and geometric mean levels of anti-gp15 IgG antibodies

Sample tested	All children (*N* = 150)		Bottled water (*N* = 73)		Municipal water (*N* = 77)	
	Number seropositive (%)	GMC (95% CI) AU	Number seropositive (%)	GMC (95% CI) AU	Number seropositive (%)	GMC (95% CI) AU
Maternal	78 (52)	465.6	42 (58)	524.1	36 (47)	405.2
		(391.3–554)		(401.3–684.6)		(326.4–503)
Child preweaning	30 (20)	342.7	19 (26)	315.9	11 (14)	394.6
		(289–406.4)		(259.4–384.6)		(278.5–559.1)
Child postinfection/end of follow-up	62 (41)	627.8	31 (42)	609.3	31 (40)	646.8
		(517.6–761.4)		(451.0–823.2)		(499.1–838.1)

AU = arbitrary units; CI = confidence interval; GMC = geometric mean concentration.

**Table 3:** Proportion of preweaning and postinfection/end-of-follow-up child seropositivity based on low, middle, and high GMC on antibodies to cryptosporidial gp15 in mothers

Maternal serological status (*n*)	Child preweaning seropositive (%)	*P* value	Child postinfection/end-of-follow-up seropositive (%)	*P* value
Seronegative (72)	9 (12.5)	–	25 (34.7)	–
Seropositive (78)	21 (26.9)	0.002	37 (47.4)	0.114
Based on GMC tertiles of seropositive mothers				
Lower (26)	3 (11.5)	0.006	10 (38.5)	0.123
Middle (26)	7 (26.9)		16 (61.5)	
Upper (26)	11 (42.3)		11 (42.3)	

GMC = geometric mean concentration.

**Table 4:** Selected serological studies with longitudinal follow-up or paired (pre- and postinfection) sampling

Study site(s)	Year	Type of study	Age group(s)	Serological method	Antigen	Key outcomes	Reference
United States*	1989	Pre/post	24–58 years	ELISA	Oocysts lysate	32% had initial detectable levels of *Cryptosporidium* IgG	31
						Seroconversion patterns for 6 weeks (5%), 1 year (14%), and 2 years (13.6%)	
						1 year group: seropositivity increased from 27% to 39% after 1 year, 41% had detectable levels at different sampling times	
						2 year group: seropositivity increased from 36% to 73% after 1 year, 82% had detectable levels at different sampling times	
Manila, Philippines	1990	Longitudinal	1–24 months	ELISA	Oocysts lysate	No increase in antibody levels after 1–6 weeks follow-up	32
Melbourne, Australia, and Goroka, Papua New Guinea	1994	Longitudinal	1–84 months	ELISA	Oocysts lysate	Antibodies peak 3–6 weeks after infection and fell to baseline levels by 6 months	21
						Seropositivity rose from 15% (< 6 months) to 64% (> 2 years) in Papua New Guinea	
						Seropositivity rose from 3% (< 6 months) to 11% (> 2 years) in Melbourne	
Texas, United States	1997	Pre/post (longitudinal sampling)	20–45 years	Immunoblot and ELISA	15/17 and 27 kDa	Fewer oocysts were excreted by volunteers with preexisting IgG antibodies to 27-kDa antigen compared with volunteers without the antibody	33
						IgG reactivity to the 17-kDa antigen was higher in asymptomatic than symptomatic infected volunteers	
						IgG reactivity to the three antigens peaked by day 32 postinoculation	
Oregon, United States	1998	Pre/post	18–60 years	Western blot	15/17 and 27 kDa	Mean antibody level after 2 years remained at 91% of the initial value for the 15/17-kDa antigen	34
						Mean antibody level after 2 years declined to 54% of the initial value for the 27-kDa antigen	
Negev, Israel	2001	Longitudinal	0–2 years	ELISA	Oocysts lysate	Infants had one-third the level of antibodies found in mothers	30
						Level of IgG antibodies dropped significantly by 6 months of age	
						Seroconversion rate of 42% to *Cryptosporidium* around 6–23 months of age	
						Seroprevalence 13% (< 5 years), 38% (5–13 years), and 58% (14–21 years)	
						*Cryptosporidium* antigen detected in 11% (6 months) and 48% (23 months)	
Texas, United States	2004	Pre/post	18–45 years	ELISA	TRAP-C1	Uninfected individuals showed higher reactivity at baseline compared with infected individuals	35
						Increase in antibody response was seen in days 30 and 45 compared with days 0 and 5	
Dhaka, Bangladesh	2004	Case–control (pre/post sampling)	≤ 5 years	ELISA	Oocysts lysate	64% seropositivity in cases and 57% in controls	36
						Significant increase in IgG levels in cases compared with controls in follow-up	
Lima, Peru	2006	Pre/post	1 month–10 years	ELISA	17 and 27 kDa	Peak antibody detection was at 15.3 and 26.7 months of age	18
						Antibody levels were higher during the second serological response	
						Antibody response increases with age and infection experience	
Dhaka, Bangladesh	2011	Case–control (pre/post sampling)	15 days–60 months	ELISA	gp15	Increase in follow-up IgG levels significantly greater in cases than controls	19
						Significant increase in IgG levels response to *Cryptosporidium parvum* and *Cryptosporidium hominis* gp15	
Vellore, India	2011	Longitudinal	Birth–3 years	ELISA	gp15	Increase in serum IgG levels after first episode of cryptosporidial diarrhea	17
						Peak response between 8 and 11 weeks (∼9 weeks) postexposure	
						Serological response to infection did not depend on baseline values	
Dhaka, Bangladesh	2012	Case–control (pre/post sampling)	15 days–60 months	ELISA	Cp23	Increase in follow-up IgG, IgM, IgA levels significantly greater in cases than controls	20
						Cases with acute diarrhea had significantly greater serum IgA and IgM responses than those with persistent diarrhea	
Vellore, India	2012	Longitudinal	0–2 years	ELISA	gp15	Seropositivity: maternal (89.2%), child at 3.5 months (31.8%), and child at 2 years (94.6%)	37
						No difference in serum IgG levels in mothers and children between cases and controls	
						76.7% remained seropositive or seroconverted at 9 months	
						Seroconversion at 9 months irrespective of the serological status of the mother	
Leogane, Haiti	2014	Longitudinal	3 weeks–11.5 years	Multiplex bead assay	17 and 27 kDa	97.9% had one serologically positive episode throughout the 10-year period	38
						28.9% had secondary *Cryptosporidium* IgG response	
						IgG responses to *Cryptosporidium* tend to increase with age	

ELISA = enzyme-linked immunosorbent assay; IgG = immunoglobulin G; “TRAP-C1 = thrombospondin-related adhesive protein of *Cryptosporidium*-1.

* Samples obtained from U.S. Peace Corps volunteers before and after service posting in western Africa.
